# Synergistic Effects of Early Psychosocial Factors and Polygenic Risk for Smoking: a Cross‐Sectional Analysis of a Sample of Older Adults in the United States

**DOI:** 10.1002/dev.70152

**Published:** 2026-03-31

**Authors:** Walter G. Dyer, Sung‐Ha Lee, Hannah Wilding, Brianna Sutara, Harold H. Lee

**Affiliations:** ^1^ Department of Psychology The Pennsylvania State University State College Pennsylvania USA; ^2^ Center for Happiness Studies Seoul National University Seoul South Korea; ^3^ The Penn State College of Medicine Hershey Pennsylvania USA; ^4^ Department of Biology The Pennsylvania State University State College Pennsylvania USA; ^5^ Department of Biobehavioral Health The Pennsylvania State University State College Pennsylvania USA

**Keywords:** early psychosocial factors, polygenic risk scores, smoking

## Abstract

Genetic influence on smoking may be modified by negative early psychosocial factors (EPFs). However, few studies have examined this interaction. We hypothesized that negative EPFs will exacerbate the association between genetic risk for smoking and ever smoking. We used data on European Americans (EA) and African Americans (AA) from the Health and Retirement Study (HRS). Polygenic score for “ever smoking” (PGS_smoking_) was assessed using PRSice from a 2010 GWAS conducted by the Tobacco and Genetics Consortium. We operationalized PGS_smoking_ as a binary variable: top 25% versus the rest. Ever smoking was assessed by asking participants if they had smoked 100 or more cigarettes in their lifetimes. EPFs included education levels of mother or father, perceived financial status, maternal warmth, and stressful events before age 18. We used logistic regression to assess the odds ratio (OR) for PGS_smoking_, EPFs, and their interaction terms in relation to ever smoking, adjusting for age, gender, and the five principal components. Among the 6969 EA participants (mean age: 74.3 years, 57% women), 55% reported ever smoking. Among the 2141 AA participants (mean age: 59.6, 67.3% women), 61.1% reported ever smoking. Within the AA sample, high genetic risk (top 25%) was associated with a 18% higher likelihood of ever smoking for AA (95% confidence interval [CI] = 0.91–1.52). Within the EA sample, high genetic risk was associated with a 25% higher likelihood of ever smoking (95% CI = 1.11–1.41), and low maternal warmth and PGS_smoking_ showed significant additive interactions to increase odds of ever smoking. The expected OR for those exposed to both high genetic risk and low maternal warmth exceeded the sum of their individual effects (relative risk due to interaction [RERI] = 0.42 [0, 0.85], *p* = 0.03), resulting in 75% higher odds of smoking compared to individuals with none of these exposures. These synergistic effects observed within the EA sample were not observed within the AA sample. Several EPFs and PGS_smoking_ were associated with increased odds of ever smoking. Among EA participants, low maternal warmth was associated with exacerbated genetic predisposition to smoking. No synergy between EPFs and genetic risk was observed for AA participants.

## 1 Introduction

Cigarette smoking is a complex behavior determined by an interaction of environmental, developmental, and genetic effects, and it is currently the leading cause of preventable death worldwide (World Health Organization 2017). Twin studies suggest that smoking initiation and persistence is 40%–60% heritable (Li 2003), and genome‐wide association studies reveal genetic variants associated with smoking behaviors (e.g., nicotine dependence and smoking initiation; Fan et al. 2021; Karlsson Linnér et al. 2019). In such genome‐wide approaches, the entire spectrum of genomic variants (i.e., ∼700,000 single‐nucleotide polymorphisms [SNPs]) is scanned with substantially large sample sizes (e.g., *n* = roughly 10,000 ‐1,000,000) to identify numerous genetic variants with minute effects on the phenotype. The genetic variants identified are aggregated into a single index of genetic risk for the phenotype and called polygenic scores (PGSs). Recent approaches to calculating PGSs, in which the score is optimized for maximal phenotypic explanations (e.g., PRSice, LDpred, and PRS‐CS; Choi and O'Reilly 2019; Privé et al. 2020), can account for a nontrivial proportion of the variance of phenotypic expressions (Belsky et al. 2013; Euesden et al. 2015; Tobacco and Genetics Consortium 2010). Compared to an SNP or PGS derived from a limited set of 5–100 SNPs, recent PGSs offer enhanced power to detect interactions between PGS with developmental factors.

Decades of social science research has accumulated robust empirical evidence highlighting the importance of early psychosocial experiences in shaping lifelong habits (Heckman 2008; McEwen 2003) including smoking (Anda et al. 1999; Ford et al. 2011). However, studies examining PGS for smoking (PGS_smoking)_ and their interaction with psychosocial factors have mostly focused on adult experiences (Pasman et al. 2019; Schmitz and Conley 2016). One investigation from Netherlands Twin Study (*n* = 1489) focused on early experiences and found that PGS_smoking_ predicted smoking heaviness for individuals exposed to cigarette smoke in childhood, but not for unexposed individuals (Treur et al. 2018). Another study focused on early experiences involving 1547 African American (AA) participants from the Detroit Neighborhood Health Study. Here, PGS_smoking_ had a stronger influence on smoking behavior among those who reported higher frequency of lifetime trauma (Meyers et al. 2013). While these studies suggest that early psychosocial factors (EPFs) may modify genetic predisposition to smoking, only a limited number of EPFs have been examined with relatively small sample sizes (e.g., *n* < 2000) for gene–environment investigations. Also, approximately 72% of genetic discoveries are from predominantly White populations from the United States, United Kingdom, and Iceland (Mills and Rahal 2019). Furthermore, although the public health burden of smoking disproportionately affects racial and ethnic minority groups, genetic research has progressed much further in White populations because non‐White individuals have been historically underrepresented in genetic studies. To fill these gaps, we examined five negative EPFs as effect modifiers for PGS_smoking_ and later self‐reported ever smoking in samples of European‐ (*n* = ∼6000) and African‐ (*n* = ∼2000) descendant older adults in the United States, and hypothesized that the presence of these factors would be associated with an exacerbation of genetic predisposition to smoking.

## 2 Methods

### 2.1 Study Sample

The Health and Retirement Study (HRS) is a nationally representative, longitudinal panel study in the United States, designed to capture comprehensive health and behavioral data for Americans over the age of 50. It surveys approximately 20,000 respondents every 2 years and introduces a new cohort every 6 years (RAND 2022; Juster and Suzman 1995). Genotype data on about 15,000 HRS participants were collected from a random subset of the 26,000 participants chosen to take part in additional in‐person visits where a saliva specimen was collected for DNA in 2006, 2008, and 2010. Our analytic sample included participants who provided both genotype data and complete data on EPFs. Out of the 6969 European Americans (EA) who provided genotype data, the rate of missing negative EPFs data ranged from 1%–20%. Among the 2141 AA who provided genotype data, the rate of missing negative EPFs data ranged from 1%–40%. The final analytic sample comprised approximately 6000 EA and 2000 AA participants, with exact counts reported in Tables 2a and 2b, respectively.

Although the PGSs used by HRS were derived from GWAS conducted in EA samples, our analyses include both EA and AA participants. Given the well‐documented reductions in predictive performance of PGSs in non‐European ancestry groups (e.g., Carlson et al. 2013; Martin et al. 2017), we characterize differences in predictive utility across these groups.

### 2.2 Measures

#### 2.2.1 Genotyping and Imputation

Saliva specimens collected in 2006, 2008, and 2010 were genotyped as part of the HRS 2012 Genetic Data Release. Participants were genotyped using two Illumina arrays: the HumanOmni2.5‐Quad BeadChip and the HumanOmni1‐Quad BeadChip. The HRS genetics team conducted standardized quality control procedures, including filters for call rate, Hardy–Weinberg equilibrium, relatedness, and batch effects. After quality control, genotypes were imputed to the 1000 Genomes Phase I (v3) reference panel using Minimac, resulting in approximately 2.4 million high‐quality imputed SNPs (Ware et al. 2017).

#### 2.2.2 PGS_smoking_ Initiation (Ever/Never)

PGS_smoking_ was constructed by HRS staff using PRSice software and summary statistics from a 2010 GWAS conducted by The Tobacco and Genetics Consortium. The discovery GWAS included 74,053 individuals across multiple cohorts, with an additional meta‐analysis of 143,023 participants examining the 15 most significant loci (Tobacco and Genetics Consortium 2010). PRSice was run using clumping parameters of *r* ^2^ = 0.1 and a 250‐kb window, and scores were calculated across a range of *p*‐value thresholds (from *p* < 5 × 10^−8^ to *p* = 1). The final PGS_smoking_ reflects the threshold that maximized variance explained within each ancestry group, as implemented by the HRS genetics team (Ware et al. 2017). PRSice calculates PGSs by using a clumping‐and‐thresholding procedure to reduce linkage disequilibrium and identify the most predictive set of SNPs for score construction.

EA PGS_smoking_ contains 710,288 SNPs that overlap between the HRS genetic database and the GWAS meta‐analysis, and AA PGSs contain 707,989 SNPs. All PGSs have been standardized within ancestry group to a standard normal curve (Ware et al. 2017). For our analyses, PGS_smoking_ was examined as a binary variable to maximize the interpretability of interaction analyses. Individuals in the upper 25th percentile of PGS_smoking_ were categorized as “high” genetic risk, and all others were categorized as “low to moderate” genetic risk for reporting ever smoking. We also ran sensitivity analyses where genetic risk was included as a continuous variable.

#### 2.2.3 Ever Smoking

Whether participants were classified as people who ever smoked was assessed via a single yes/no question, “Have you smoked 100 or more cigarettes in your life?” from Wave 1 (1992) until Wave 13 (2016). This inclusive approach ensured that participants who endorsed this item at any wave were classified as people who ever smoked.

#### 2.2.4 Exposures

To facilitate cross‐national comparison in the future, we employed EPFs from the Harmonized HRS dataset's “Childhood/Lifetime Stressful Events” section (version C [1992–2019], January 2022): (1) maternal warmth, (2) perceived financial status, (3) number of childhood stress events, (4) paternal education, and (5) maternal education.

1. Maternal warmth (low vs. normal) was dichotomized as low versus high. This variable was calculated by summing the following three components: “How much time and attention the respondent's mother gave them when they needed it before age 18” (1 [not at all] to 4 [a lot]), “how much effort the respondent's mother put into watching over and making sure the respondent had a good upbringing before age 18” (1 [not at all] to 4 [a lot]), and “how much the respondent agrees that they had a good relationship with their mother before age 18,” (1 [strongly disagree] to 5 [strongly agree]). From these, the maternal mean score (range: 1–4, median: 3) lower than the bottom 25% was considered “low,” with the rest as “normal”.
2. Perceived financial status during childhood (high vs. normal) is the respondent's self‐rated family financial situation while they were growing up before age 16. The respondents who perceived their childhood financial situation to be or “poor” were categorized as “high”, and those who reported “pretty well off” or “about average” were categorized as “normal”.
3. Childhood stress events assessed whether participants experienced a stressful event by the age of 18 (yes vs. no) based on (1) whether the respondent reported police involvement, (2) whether either of the respondent's parents drank or used drugs so often that it caused family problems, or (3) whether the respondent was physically abused by either of their parents before age 18.

(4/5) Paternal/maternal education (low vs. normal) was assessed based on whether the respondents reported that their father/mother, respectively, attained an education of more than 8 years.

Gender was determined by a single self‐report item where respondents reported whether they identify as male and female. Age was determined by participants’ birth dates. The first five principal components of ancestry, provided by HRS, were employed to account for potential population stratification and possible ancestry differences in genetic background. Gender, age, and the first five principal components were included as covariates for all analyses.

### 2.3 Statistical Analysis

Multivariate logistic regression models were employed to examine multiplicative and additive interactions between PGS_smoking_ (genetic exposure) and negative EPFs (environmental exposure) on ever smoking (outcome) for the five negative EPFs separately, adjusting for gender and the five principal components. Consistent with recommendations for presenting interaction analyses (Knol and VanderWeele 2012), we calculated odds ratios (ORs) and 95% confidence intervals (CI) of both multiplicative and additive interaction (i.e., relative risk due to interaction [RERI]) and the ORs of genetic and psychosocial exposure, respectively, controlling for gene–EPF interaction and the covariates. A RERI greater than zero indicated a positive deviation from additivity, and it was considered significant when the 95% CI did not include zero, with RERIs calculated using the delta method based on model‐derived ORs. Controlling for the same covariates, we also performed stratified analyses by both genetic and psychosocial exposures. Survey weights were not applied because the ever‐smoking variable covers the entire lifespan over and beyond the follow‐up period in HRS (i.e., 1992–2018). All analyses were conducted using R (R Foundation for Statistical Computing 2016), and the code can be found online at: https://osf.io/fhjvm/.

## 3 Results

### 3.1 Sample Characteristics

Sample characteristics are summarized in Table 1. EA were, on average, older than AA. Just over half of EA and AA smoked, and smoking rates were higher among AA relative to EA. Negative EPFs did not differ by EA and AA. A link between age and smoking was observed, such that a 10‐year increase in age was associated with 5.6% lower odds of ever smoking (*p* < 0.05).

**TABLE 1:** Characteristics of participants (2016) by self‐reported race/ethnicity.

Characteristics	Race/ethnicity	
	African American	European American
	(*n* = 2141)	(*n* = 6969)
Sample characteristics		
Ever smoking (%)	1275 (59.6)	3838 (55.1)
Mean age, years (SD)	67.3 (11.0)	74.3 (14.3)
Women (%)	61.1	57.2
Psychosocial factors		
Mean number of childhood stress events (SD)	0.3 (0.6)	0.3 (0.6)
Mean maternal warmth (SD)^a^	2.9 (0.5)	2.9 (0.4)
Paternal education > 8 (%)	886 (64.3)	5784 (83.0)
Maternal education > 8 (%)	1384 (76.6)	6209 (89.1)
Child finance (%)^b^		
“Pretty well‐off”	139 (6.5)	525 (8.5)
“About average”	1167 (54.8)	4160 (67.3)
“Poor”	824 (38.7)	3597 (57.4)

*Note:* Percentage may not add up to 100% due to missing values.

^a^Maternal warmth is based on the earliest report of (1) how much time and attention the respondent's mother gave them when they needed it before age 18, (2) how much effort the respondent's mother put into watching over and making sure the respondent had a good upbringing before age 18, and (3) how much the respondent agrees that they had a good relationship with their mother before age 18.

^b^Child finance is the respondent's self‐rated family financial situation while they were growing up.

### 3.2 PGS_smoking_

High genetic risk (top 25%) was associated with a 25% higher likelihood of ever smoking (95% CI = 1.11–1.41) for EA and 18% higher likelihood of ever smoking for AA (95% CI = 0.91–1.52).

### 3.3 Ever Smoking in a Lifetime: Genetic Risk for Smoking and Early Psychological Factors

**FIGURE 1:**
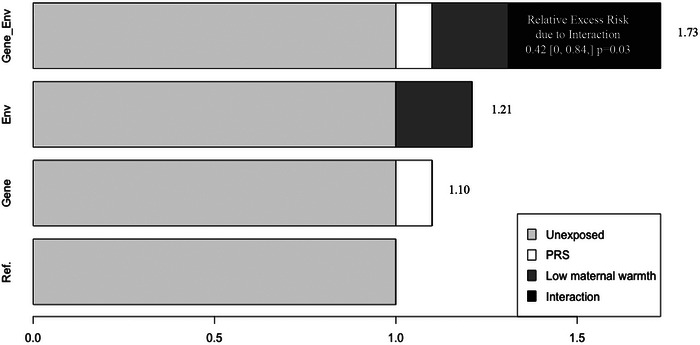
Additive effects of maternal warmth and the polygenic risk score for ever smoking, 75% cut‐point (PGS), on ever smoking (yes/no) adjusted for gender and five principal components; RERI—relative excess risk due to interaction (*n* = 5572) among the European American sample.

Tables 2a and 2b present a comprehensive overview for EA and AA samples, respectively, of the (1) calculated ORs and corresponding 95% CI for both genetic and psychosocial exposures, accounting for their potential interactions; (2) stratified analyses by both genetic and psychosocial exposures; and (3) interaction analyses by the multiplicative scale and RERI. Among EA, controlling for the interaction terms with each negative EPF, individuals with a high genetic risk for smoking exhibited greater odds of ever having smoked, with ORs spanning the range of 1.10–1.32 (Table 2a). When controlling for the interaction with the genetic risk of smoking, exposure to childhood stress events was associated with a 72% increase in odds of ever smoking (OR = 1.72, 95% CI = 1.50–1.98), and lower levels of maternal warmth were linked to a 25% elevated likelihood of ever smoking (OR = 1.25, 95% CI = 1.06–1.41). Notably, interaction analyses revealed that maternal warmth and genetic risk for smoking may have additive interactions to influence smoking behavior (RERI = 0.42 [0, 0.85], *p* = 0.03). Figure 1 shows the expected OR for those exposed to both genetic and psychosocial factors exceeding the sum of their individual effects, resulting in 75% higher odds of smoking compared to individuals with none of these exposures among the EA sample. This interaction was not observed with other psychosocial factors. Among AA, none of the negative EPFs tested significantly modified the PGS_smoking_–ever smoking association (Table 2a). In analyses where each psychological factor was entered into a separate model as an interaction term with the continuous smoking PGS, the smoking PGS × maternal warmth term emerged as the sole statistically significant interaction in the EA sample (*p* = 0.02) (Table 2a), and none of the interaction terms were statistically significant within the AA sample (*p* > 0.05) (Table 2b).

**TABLE 2a:** Interaction of polygenic risk score of smoking and early psychosocial experience on ever smoking in European Americans.

	PGS‐smoking < 75th percentile (0)		PGS‐smoking > 75th percentile (1)		OR^a^ of PGS‐smoking within psychosocial strata	Interaction analyses
	*N* (cases/controls)	OR^a^ (95% CI)	*N* (cases/controls)	OR^a^ (95% CI)		
High maternal warmth (0)	1574/3063 (51%)	1.00 (reference)	547/1004 (55%)	1.10 (0.95, 1.28)	1.10 (0.95, 1.28)	*N* = 5572 Multiplicative scale: 1.3 (0.98, 1.72), *p* = 0.07 RERI: 0.42 (0, 0.84), ***p* = 0.03**
Low maternal warmth (1)	629/1120 (56%)	1.21 (1.05, 1.39)	248/385 (64%)	1.73 (1.37, 2.17)	1.43 (1.11, 1.83)	
OR^a^ (95% CI) of the psychosocial factor within genetic strata		1.21 (1.05, 1.39)		1.57 (1.22, 2.01)		
Low childhood financial burden (0)	905/1549 (58%)	1.00 (reference)	2517/4717 (53%)	1.23 (1.08, 1.39)	1.23 (1.08, 1.39)	*N* = 6868 Multiplicative scale: 1.25 (0.84, 1.85), *p* = 0.28 RERI: 0.39 (−0.24, 1.03), *p* = 0.11
High childhood financial burden (1)	111/168 (66%)	1.14 (0.93, 1.40)	247/434 (57%)	1.75 (1.25, 2.44)	1.53 (1.05, 2.23)	
OR^a^ (95% CI) of the psychosocial factor within genetic strata		1.14 (0.93, 1.40)		1.45 (1.01, 2.00)		
Low childhood stress events (0)	632/1138 (56%)	1.00 (reference)	1719/3488 (49%)	1.27 (1.1, 1.47)	1.27 (1.1, 1.47)	*N* = 6187 Multiplicative scale: 0.93 (0.7, 1.22), *p* = 0.58 RERI: 0.04 (−0.47, 0.53), *p* = 0.44
High childhood stress events (1)	275/410 (67%)	1.71 (1.48, 1.97)	731/1151 (64%)	2.02 (1.61, 2.52)	1.18 (0.92, 1.51)	
OR^a^ (95% CI) of the psychosocial factor within genetic strata		1.71 (1.48, 1.97)		1.58 (1.24, 2.02)		
High father education (0)	2002/3831 (52%)	1.00 (reference)	753/1275 (59%)	1.32 (1.15, 1.51)	1.32 (1.15, 1.51)	*N* = 6151 Multiplicative scale: 0.86 (0.63, 1.18), *p* = 0.36 RERI: −0.17 (−0.55, 0.21), *p* = 0.81
Low father education (1)	408/780 (52%)	1.09 (0.92, 1.28)	148/265 (56%)	1.24 (0.95, 1.61)	1.14 (0.85, 1.52)	
OR^a^ (95% CI) of the psychosocial factor within genetic strata		1.09 (0.92, 1.28)		0.94 (0.71, 1.24)		
High mother education (0)	2315/4348 (53%)	1.00 (reference)	839/1426 (59%)	1.26 (1.10, 1.43)	1.26 (1.10, 1.43)	*N* = 6479 Multiplicative scale: 0.99 (0.68, 1.42), *p* = 0.94 RERI: 0.02 (−0.47, 0.51), *p* = 0.47
Low mother education (1)	275/518 (53%)	1.15 (0.94, 1.39)	110/187 (59%)	1.42 (1.04, 1.94)	1.24 (0.88, 1.76)	
OR^a^ (95% CI) of the psychosocial factor within genetic strata		1.15 (0.94, 1.39)		1.13 (0.82, 1.56)		

*Note:* Bold values represent statistically significant OR with *p* < 0.05.

Abbreviations: CI, confidence interval; N, number of subjects; OR, odds ratio; PGS, polygenic risk score.

^a^ ORs (95% CIs) estimated by logistic regression models with ever smoking (yes/no) as the outcome, adjusted for age, gender, and five principal components. In analyses where each psychological factor was entered into a separate model as an interaction term with the continuous smoking PGS, the smoking PGS × maternal warmth term emerged as the sole statistically significant interaction (*p* = 0.02).

**TABLE 2b:** Interaction of polygenic risk score of smoking and early psychosocial experience on ever smoking in African Americans.

	PGS‐smoking < 75th percentile (0)		PGS‐smoking > 75th percentile (1)		OR^a^ of PGS‐smoking within psychosocial strata	Interaction analyses
	*N* (cases/controls)	OR^a^ (95% CI)	*N* (cases/controls)	OR^a^ (95% CI)		
High maternal warmth (0)	417/738 (57%)	1.00 (reference)	168/268 (63%)	1.28 (0.90, 1.82)	1.28 (0.90, 1.82)	*N* = 1368 Multiplicative scale: 0.85 (0.42, 1.76), *p* = 0.67 RERI: −0.15 (−1.20, 0.90), *p* = 0.61
Low maternal warmth (1)	180/287 (63%)	1.41 (1.00, 1.98)	50/75 (67%)	1.54 (0.86, 2.77)	1.09 (0.58, 2.05)	
OR^a^ (95% CI) of the psychosocial factor within genetic strata		1.41 (1.00, 1.98)		1.20 (0.64, 2.27)		
Low childhood financial burden (0)	869/1502 (58%)	1.00 (reference)	308/489 (63%)	1.21 (0.92, 1.58)	1.21 (0.92, 1.58)	*N* = 2130 Multiplicative scale: 0.82 (0.31, 2.17), *p* = 0.69 RERI: −0.21 (−1.47, 1.04), *p* = 0.63
High childhood financial burden (1)	63/95 (66%)	1.31 (0.77, 2.22)	27/44 (61%)	1.30 (0.59, 2.87)	0.99 (0.39, 2.53)	
OR^a^ (95% CI) of the psychosocial factor within genetic strata		1.31 (0.77, 2.22)		1.08 (0.48, 2.43)		
Low childhood stress events (0)	458/837 (55%)	1.00 (reference)	169/282 (60%)	1.15 (0.81, 1.63)	1.15 (0.81, 1.63)	*N* = 1482 Multiplicative scale: 1.43 (0.69, 2.94), *p* = 0.33 RERI: 0.67 (−0.61, 1.95), *p* = 0.15
High childhood stress events (1)	183/276 (66%)	1.28 (0.90, 1.81)	64/87 (74%)	2.1 (1.17, 3.77)	1.64 (0.87, 3.09)	
OR^a^ (95% CI) of the psychosocial factor within genetic strata		1.28 (0.90, 1.81)		1.82 (0.97, 3.44)		
High father education (0)	527/890 (59%)	1.00 (reference)	198/339 (58%)	1.05 (0.14, 7.79)	1.05 (0.14, 7.79)	*N* = 1277 Multiplicative scale: 0.77 (0.14, 4.11), *p* = 0.76 RERI: −0.3 (−2.48, 1.88), *p* = 0.61
Low father education (1)	24/41 (59%)	1.19 (0.51, 2.76)	4/7 (57%)	1.14 (0.48, 2.73)	0.96 (0.71, 1.32)	
OR^a^ (95% CI) of the psychosocial factor within genetic strata		1.19 (0.51, 2.76)		1.09 (0.17, 6.91)		
High mother education (0)	716/1210 (59%)	1.00 (reference)	250/407 (61%)	2.33 (0.63, 8.57)	2.33 (0.63, 8.57)	*N* = 1699 Multiplicative scale: 0.5 (0.13, 1.88), *p* = 0.30 RERI: −1.14 (−4.14, 1.87), *p* = 0.77
Low mother education (1)	33/56 (59%)	1.24 (0.66, 2.32)	18/26 (69%)	1.43 (0.74, 2.77)	1.16 (0.87, 1.54)	
OR^a^ (95% CI) of the psychosocial factor within genetic strata		1.24 (0.66, 2.32)		0.61 (0.19, 1.99)		

*Note:* Bold values represent statistically significant OR with *p* < 0.05.

Abbreviations: CI, confidence interval; N, number of subjects; OR, odds ratio; PGS, polygenic risk score.

^a^ ORs (95% CIs) were estimated by using logistic regression model with ever smoking (yes/no) as the outcome, adjusted for gender and five principal components. In analyses where each psychological factor was entered into a separate model as an interaction term with the continuous smoking PGS, none of the interaction terms were statistically significant (*p* > 0.05).

## 4 Conclusions

Our findings suggest that those with heightened genetic risk for smoking were more likely to report ever smoking than those with low genetic risk. Among the AA sample, none of the negative EPFs modified the PGS_smoking_–ever smoking association. Among the negative EPFs tested in the EA sample, childhood stress and low maternal warmth were associated with higher odds of smoking, controlling for gene–EPF interaction. Within this sample, we observed a significant interaction between low maternal warmth and genetic risk, such that having both risk factors increased the odds of ever smoking beyond the sum of the effects of each exposure variable alone.

Our findings align with nascent literature on this topic. For example, our null finding concerning parental education is consistent with a prior study of 20,895 Dutch adults, which also found that educational attainment does not modify genetic influence on smoking (Pasman et al. 2022). Our findings regarding low maternal warmth are in line with longitudinal data from 3929 middle‐aged Americans, demonstrating that high parental warmth is associated with a reduced likelihood of smoking (Chen et al. 2019). We extend this finding by showing that low maternal warmth is linked to an exacerbation of genetic predisposition to smoking. For populations with a high genetic susceptibility to smoking, a plausible preventive strategy may be intervening during childhood/adolescence (i.e., early intervention), considering robust and large empirical evidence highlighting the developmental advantages and cost‐effectiveness of fostering lifelong healthy habits during this critical developmental period (García and Heckman 2021; Heckman 2006; Knudsen et al. 2006). PGS_smoking_ was a stronger predictor of ever smoking in the EA sample. Moreover, the exacerbation of this association under conditions of low maternal warmth was observed only among EA. These differences between the EA and AA samples may reflect the fact that most GWAS to date have been conducted primarily with European ancestry populations. We encourage future GWAS research that addresses this disparity via focusing on underrepresented groups.

Some limitations of our study should be maintained when interpreting our findings. First, “ever smoking” is a relatively shallow phenotype when assessing genetic risk for smoking. Recent GWASs have calculated PGS for physiological risk factors for nicotine addiction, such as nicotine metabolism speed (Hukkanen et al. 2005), which enables researchers to examine deeper smoking phenotypes (Xu et al. 2020). Second, we were limited in the types of EPFs we could examine. Third, we examined effect modification of PGS_smoking_ on “ever smoking” which likely overlooks some of the nuanced effects of genetic risk for smoking. Accordingly, we encourage future investigations of PGS_smoking_ that include more detailed information regarding smoking status as the outcome—such as never/current/and prior smoking—possibly via the implementation of a time‐varying effect model (e.g., Wilding et al. 2025). Lastly, because the sample consisted of Americans over the age of 50, the findings may not generalize to younger US populations or to populations outside the United States.

In conclusion, we found that the combined effects of low maternal warmth and high genetic risk for smoking synergistically increased the risk of cigarette smoking beyond the added individual effects of these factors alone. These effects were observed among EA but not AA. For populations with high genetic risk for smoking, interventions aimed at increasing maternal warmth may buffer the genetic effect on smoking.

## Funding

The HRS is sponsored by the National Institute on Aging (grant number NIA U01AG009740) and is conducted by the University of Michigan. Walter G. Dyer was supported by the National Institutes of Health's Prevention and Methodology Training Program (T32DA017629, principal investigators J. Maggs and S. Lanza) with funding from the National Institute of Drug Abuse. The content is solely the responsibility of the authors and does not represent the official views of the National Institute on Drug Abuse or National Institute on Aging.

## Ethics Statement

The study was deemed exempt by the Penn State University Institutional Review Board because the data were de‐identified.

## Consent

The authors have nothing to report.

## Conflicts of Interest

The authors declare no conflicts of interest.

## Data Availability

This dataset is publicly available on the Health and Retirement Study (HRS) website.
